# Understanding antibiotic use and antimicrobial resistance: A cross-sectional study among outpatients in Greek primary health care

**DOI:** 10.3934/publichealth.2026033

**Published:** 2026-05-22

**Authors:** Aikaterini Toska, Dimitra Kourneta, Dimos Mastrogiannis, Evangelos Fradelos, Pavlos Sarafis, Eustratia Mourtou, Marianna Mantzorou, Maria Saridi

**Affiliations:** 1 Department of Nursing, University of Thessaly, Greece, Open Hellenic University, Greece; 2 Open Hellenic University, Greece; 3 Department of Nursing, University of West Attica, Athens, Greece; 4 Department of Information Systems, Informatics Directorate, 6th Ministry of Education, Open Hellenic University, Greece

**Keywords:** antimicrobial resistance, antibiotic use, knowledge, attitudes, outpatients, healthcare setting

## Abstract

**Background:**

Antibiotics are crucial for treating bacterial illnesses; however, overuse and abuse of these drugs, together with improper consumption, have led to selection pressure and the rise of resistant bacteria.

**Aim:**

To investigate the knowledge and attitudes of outpatients in a healthcare setting regarding antibiotic consumption and antimicrobial resistance.

**Methods:**

This exploratory survey was conducted among 120 outpatients visiting a public primary healthcare setting in Greece, from February to March 2024. Data were collected via a 38-item self-administered questionnaire covering three domains: antimicrobial resistance (AMR) knowledge, antibiotic knowledge, and consumption practices. Statistical analysis was performed using SPSS (ver.29), employing Mann–Whitney and Kruskal–Wallis tests to identify demographic correlates (p < 0.05).

**Results:**

A total of 120 individuals participated in the study, 72 of whom were women (60.0%); 37.5% resided in an urban center, and 30.8% were lyceum graduates. Most of the sample knew that AMR signifies resistance of microbes to antibiotics (75.0%), that it constitutes a significant public health problem in the country (70.0%), and that it is due to the inappropriate use of antibiotics in humans (67.5%). 74.2% disagreed with stopping antibiotic treatment earlier, while 40.0% of respondents stated that taking antibiotics is only useful for fighting bacterial infections. 27.5% thought antibiotics were necessary for viral illnesses with fever, and 35.8% thought they speed up recovery from a cold. Remarkably, 52.5% of respondents acknowledged keeping antibiotics for later use, and 56.7% reported using non-prescription antibiotics in the past. Antibiotic knowledge scores were statistically significantly higher for women and people living in urban and semi-urban areas (p < 0.05). People with less education knew less about antibiotics, antimicrobial resistance, and antibiotic consumption practices (p < 0.005).

**Conclusions:**

Although primary health care recipients demonstrate a generally satisfactory awareness of antimicrobial resistance, significant knowledge gaps and misconceptions about antibiotics persist, leading to inappropriate antibiotic use practices.

## 1. Introduction

Antimicrobial resistance (AMR) is one of the most serious and growing threats to public health worldwide, with the COVID-19 pandemic influencing its dynamics [1], causing significant clinical, social, and economic impacts [2],[3]. The spread of resistant microorganisms leads to prolonged hospitalizations, increased mortality rates [4], and high costs for healthcare systems, which in the European Union amounts to approximately 1.5 billion euros annually due to direct healthcare costs and lost productivity [5].

The relationship between antibiotic consumption and the development of antimicrobial resistance (AMR) is well documented [6],[7]. Antibiotics are crucial for treating bacterial illnesses; however, overuse and abuse of these drugs, together with improper dosage and duration, have led to selection pressure and the rise of resistant bacteria. The public contributes to the perpetuation of the phenomenon of AMR, mainly due to misconceptions arising from the lack of sufficient knowledge about antibiotics, their indications for use, and the consequences of their abuse. This ignorance leads to inappropriate consumption practices, exacerbating the problem of antimicrobial resistance [8].

The misuse of antibiotics, such as prescribing them for viral infections, pressuring doctors to prescribe, or storing antibiotics for future use, is associated with worsening resistance. Despite efforts to reduce the overuse of antimicrobials, community-level antibiotic use in the EU/EEA remains alarmingly high, ranging from 9 Defined Daily Doses (DDD)/1000 inhabitants/day in the Netherlands to 27.8 in Greece, which consistently ranks among the countries with the highest antibiotic consumption in Europe [9]. According to data collected in Greece and sent to the European Centre for Disease Prevention and Control (ECDC), total consumption of antibiotics for systemic use in Greece has been fluctuating in the past five years (26.4 DDD/1000 inhabitants/day in 2020, 21.8 in 2021, 31.2 in 2022, 26.7 in 2023, and 27.8 in 2024); nevertheless, all values are among the highest between the 25 current European Union member states [9]. This phenomenon requires a better understanding of the pathogens' resistance profile in order to plan effective treatment strategies and prevent their spread [10].

Even though 84% of Europeans recognize that the irrational use of antibiotics leads to a decrease in their effectiveness, only 43% know that antibiotics are ineffective against viruses [11], indicating significant gaps in understanding basic concepts. Population awareness and education are fundamental factors in combating AMR, as the general population plays a decisive role through its consumer behavior. As mentioned earlier, the phenomenon of applying pressure on physicians is also present in Greece, where it has been established that even parents demand physicians to prescribe antibiotics for their children. Although only a small portion of physicians give in to parents' demands (19%), this kind of practice may adversely affect AMR [12].

In order to identify widespread misconceptions and specific knowledge gaps, this study intends to explore the degree of outpatient awareness of antimicrobial resistance (AMR) and their antibiotic use behaviors. The results can guide focused educational initiatives and public health tactics to encourage more responsible antibiotic use in primary care settings by identifying these misconceptions.

## 2. Materials and methods

The present study aims to investigate the knowledge and practices of outpatients who visited a public primary health care center within the 6th Greek Health District, regarding the consumption of antibiotic drugs and microbial resistance. A convenience sampling method was adopted. To achieve the study's aim, an exploratory descriptive non-experimental survey was conducted among outpatients visiting the Health Center. Initially, permission for the study was obtained from the 6th Greek Health District.

Two authors (AT and DK) created a structured questionnaire based on worldwide literature [13],[14] to evaluate two important domains in this study: habits linked to antibiotic consumption and knowledge of the indications and efficacy of antibiotics. This instrument was divided into three separate sections to offer a thorough assessment of the study objectives and was modified from proven World Health Organization (WHO) tools to guarantee internal validity:

Eleven items in Section 1: Knowledge Regarding AMR assessed knowledge of antibiotic resistance, including its causes, transmission, and the extent to which it affects public health. One point was awarded for each right answer and zero points for “Incorrect” or “I don't know” responses to generate a knowledge score. The total score ranged from 0 to 10.

Participants' knowledge of antibiotic indications, awareness of side effects and resistance, and attitudes about clinical procedures and the doctor-patient relationship were all evaluated in Section 2, which consisted of 15 items. Section 3: Antibiotic Consumption Practices comprised 12 questions that assessed behaviors during the preceding 12 months. The overall score ranged from 0 to 15. The frequency of use, the source of the drug (prescription vs. over the counter), and the habit of hoarding leftover antibiotics were all particularly highlighted. The prevalence of self-medication was ascertained by analyzing these items as categorical data; higher values indicated greater health literacy. The range of the overall score was 0 to 1.

To evaluate the internal consistency of the three parts of the questionnaire, the Cronbach's alpha coefficient was calculated, as well as the Spearman–Brown Coefficient. To obtain a total score for every part of the questionnaire, each correct answer received a value of 1, while wrong and no answers received a value of zero, according to Geta and Kibret's methodology [13]. The total score was the sum of all correct answers. Furthermore, for the study, participants were divided into two categories: those with good knowledge about antimicrobial resistance, antibiotics, and their consumption practices, and those with less good knowledge. The criterion for their separation was the mean score they obtained in each section of the questionnaire. Specifically, individuals who scored higher than or equal to the mean value were perceived as having high levels of knowledge, while individuals who scored lower than the mean value were perceived as having low levels of knowledge. This method was also used in the article by Geta and Kibret [13].

The study lasted from February to March 2024 and included patients who visited emergency departments for health problems as well as for medicine prescriptions. 203 adult outpatients in total were first asked to participate in the survey. 83 of them chose not to take part, claiming things like lack of interest or time restrictions. A response rate of 59.1% was obtained from the collection of 120 completed responses. In the healthcare facility's waiting area, participants independently completed the survey. The researcher was only there to distribute the forms, pick them up when they were finished, and handle any logistical questions, making sure the interviewer kept the answers private and objective. Exclusion criteria specifically disqualified healthcare professionals (such as doctors, nurses, and pharmacists), medical students, people under the age of 18, people with cognitive impairments, and people who had trouble speaking or writing in order to ensure that the results accurately reflected public knowledge. The sample size was justified using a power analysis for a 95% confidence interval and a 9% margin of error; a minimum of 118 participants were required based on a 50% response distribution. Our final sample of 120 people met these statistical criteria.

Following questionnaire collection, data were coded and inserted into the statistical package SPSS (ver.29). The mean, standard deviation, and minimum/maximum values were used for the descriptive analysis of the quantitative variables, while the relative frequencies and percentages were used for the descriptive analysis of the categorical variables. The Kolmogorov–Smirnov test was used to examine whether quantitative variables followed a normal distribution. For the comparison of the mean values of quantitative variables in two groups of independent samples, the nonparametric Mann–Whitney test was used, while for the comparison of the mean values of quantitative variables between three or more groups, the nonparametric Kruskal–Wallis test was used.

Furthermore, to investigate the type and intensity of the relationship between two or more quantitative variables, the significance test for the Spearman correlation coefficient was used. Significance levels were two-tailed, and the statistical significance was set at 0.05.

### 2.1. Ethics

The study received approval from the 6th Health Region (Protocol approval number 14867/2024) on 27 February 2024.

Participation in completing the questionnaires was completely voluntary, and the anonymity and confidentiality of the participants' data were fully ensured.

## 3. Results

### 3.1. Descriptive statistical analysis

#### 3.1.1. Demographics

A total of 120 people participated, 72 of whom were women (60.0%), and the remaining 48 (40.0%) were men. The average age of the participants was 46.12 years, with the youngest being 19 years old and the oldest being 78 years old. Most of the participants fell in the 40–60 years old age group, while 64.2% stated that they were married.

Regarding the place of residence of the participants, 37.5% of the sample resided in an urban center, and 30.8% were lyceum graduates. Finally, 45.8% of the sample was employed in the public sector, while the lowest percentage (8.3%) corresponded to freelancers (Table 1).

**Table 1.** Sample's demographic characteristics.

	Ν	%
Gender		
Female	72	60.0
Male	48	40.0
Age group		
19–39	38	31.7
40–60	64	53.3
>60	18	15.0
Marital status		
Married	77	64.2
Unmarried	29	24.2
Cohabitating	5	4.2
Divorced	9	7.5
Area of Residence		
Urban center	45	37.5
Rural	45	37.5
Semi-urban	30	25.0
Level of education		
Primary school	9	7.5
High school	11	9.2
Lyceum	37	30.8
Vocational Training Institute (IEK)	14	11.7
Technological Educational Institute (TEI)	24	20.0
University	25	20.8
Profession		
Civil servant	55	45.8
Private sector employee	19	15.8
Freelancer	10	8.3
Farmer	22	18.3
Other	14	11.7

#### 3.1.2. Knowledge about antimicrobial resistance

The first part of the questionnaire was highly internally consistent, yielding a Cronbach's alpha value of 0.805. Also, the Spearman–Brown coefficient was satisfactory, yielding a value of 0.727. The results presented in Table 2 show that most of the sample had knowledge of the concept of antimicrobial resistance (84.2%) and knew that it corresponds to resistance of microbes to antibiotics (75.0%) and constitutes a significant public health problem both in the country (70.0%) and worldwide (65.0%).

Regarding the cause of antimicrobial resistance, most of the sample stated that it is due to the inappropriate use of antibiotics in humans (67.5%) and to patients taking antibiotics without medical advice (68.3%).

Regarding the transmission of antimicrobial resistance, most of the respondents stated that it cannot be transmitted from person to person and from animals to humans (49.2% and 50.8%, respectively). Finally, their information about antimicrobial resistance came mainly from television and the internet (36.7% and 24.2%, respectively).

**Table 2.** Knowledge about antimicrobial resistance.

	Yes/correct	No/wrong	I don't know/I don't wish to answer	
	Ν (%)	Ν (%)	Ν (%)
1. Have you ever heard of antimicrobial resistance?	101 (84.2)	16 (13.3)	3 (2.5)
2. Antimicrobial resistance is the resistance of microbes to antibiotics	90 (75.0)	6 (5.0)	24 (20.0)
3. Antimicrobial resistance is the resistance of humans to microbes	35 (29.2)	55 (45.8)	30 (25.0)
4. Antimicrobial resistance is a major public health problem in our country	84 (70.0)	7 (5.8)	29 (24.2)
5. Antimicrobial resistance is a major public health problem worldwide	78 (65.0)	9 (7.5)	33 (27.5)
6. Antimicrobial resistance is due to the inappropriate use of antibiotics in humans	81 (67.5)	16 (13.3)	23 (19.2)
7. Antimicrobial resistance is due to the misuse of antibiotics in livestock	26 (21.7)	42 (35.0)	52 (43.3)
8. Antimicrobial resistance is caused by patients taking antibiotics without medical advice	82 (68.3)	19 (15.8)	19 (15.8)
9. Antimicrobial resistance can be transmitted from person to person	21 (17.5)	59 (49.2)	40 (33.3)
10. Antimicrobial resistance can be transmitted from animals to humans	17 (14.2)	61 (50.8)	42 (35.0)
11. I was informed about antimicrobial resistance by:

Television	Internet	Pharmacist	Doctor	Newspaper	Other
Ν (%)	Ν (%)	Ν (%)	Ν (%)	Ν (%)	Ν (%)

44 (36.7)	29 (24.2)	5 (4.2)	20 (16.7)	6 (5.0)	16 (13.3)

#### 3.1.3. Knowledge about antibiotics

Internal consistency of the second part of the questionnaire was acceptable, yielding a Cronbach's alpha value of 0.662. Spit-half reliability was marginally acceptable, with a Spearman–Brown coefficient of 0.644. Most participants (94.2%) knew that antibiotics can cause side effects in the human body (such as diarrhea). In addition, 74.2% disagreed with stopping antibiotic treatment earlier, while for most respondents, taking antibiotics is only useful for fighting infections caused by bacteria (40.0%), and mild infections can be resolved on their own without taking antibiotics (69.2%) (Table 3).

**Table 3.** Knowledge about antibiotics.

	Correct	Wrong	I don't know/I don't wish to answer
	Ν (%)	Ν (%)	Ν (%)
1. Antibiotics can cause side effects in humans, such as diarrhea	113 (94.2)	2 (1.7)	5 (4.2)
2. Antibiotics cause negative effects on the body's normal flora	83 (69.2)	11 (9.2)	26 (21.7)
3. If someone feels better after taking antibiotics, they can stop treatment earlier	16 (13.3)	89 (74.2)	15 (12.5)
4. The use of antibiotics in animals can reduce the effectiveness of antibiotic treatment for humans	23 (19.2)	32 (26.7)	65 (54.2)
5. Any viral infection with fever should be treated with antibiotics	33 (27.5)	64 (53.3)	23 (19.2)
6. Antibiotics are only useful for fighting infections caused by bacteria	48 (40.0)	25 (20.8)	47 (39.2)
7. Mild infections may resolve on their own without antibiotics	83 (69.2)	10 (8.3)	27 (22.5)
8. Original antibiotics are more effective than generic ones	42 (35.0)	36 (30.0)	42 (35.0)
9. Expensive antibiotics are more effective than cheaper ones	15 (12.5)	66 (55.0)	39 (32.5)
10. It is not right to keep antibiotics in the cupboard to use them for future infections	78 (65.0)	32 (26.7)	10 (8.3)
11. Antibiotics should always be prescribed for infections caused by viruses	35 (29.2)	45 (37.5)	40 (33.3)
12. Ear infections always require antibiotics	43 (35.8)	46 (38.3)	31 (25.8)
13. Only doctors are responsible for the correct use of antibiotics	109 (90.8)	10 (8.3)	1 (0.8)
14. Patients should not pressure doctors to prescribe antibiotics	112 (93.3)	8 (6.7)	0 (0.0)
15. Doctors should take the time to explain to their patients why they should or should not prescribe antibiotics	116 (96.7)	2 (1.7)	2 (1.7)

#### 3.1.4. Practices regarding the consumption of antibiotics

Internal consistency of the third part of the questionnaire was acceptable, yielding a Cronbach's alpha value of 0.681. The Spearman–Brown coefficient had a satisfactory value of 0.741. Our data showed that most participants took their antibiotics according to the doctor's instructions (95.8%) without pressuring them to prescribe antibiotics in case they disagreed (85.0%), while a percentage of 75.0% would not stop taking their treatment when they felt better. However, 56.7% of the sample had taken antibiotics without a doctor's prescription in the past, and 52.5% of participants kept antibiotics in their cupboard for future use. Finally, 70.8% were concerned about the impact that antibiotic resistance could have on their health and the health of their family (Table 4).

**Table 4.** Practices regarding the consumption of antibiotics.

	Yes	No	I don't know/I don't wish to answer
	Ν (%)	Ν (%)	Ν (%)
1. Antibiotics help me recover from a cold much faster	43 (35.8)	67 (55.8)	10 (8.3)
2. I ask for antibiotics even if my doctor advises me not to take them	18 (15.0)	102 (85.0)	0 (0.0)
3. I have pressured my doctor to prescribe antibiotics for me even if he/she disagreed	25 (20.8)	94 (78.3)	1 (0.8)
4. I have bought antibiotics in the past from the pharmacy without a prescription	68 (56.7)	52 (43.3)	0 (0.0)
5. I take my antibiotics as recommended by my doctor	115 (95.8)	5 (4.2)	0 (0.0)
6. I take extra antibiotic pills if my cold gets worse	31 (25.8)	84 (70.0)	5 (4.2)
7. I stop taking my antibiotic when I feel better	28 (23.3)	90 (75.0)	2 (1.7)
8. I share antibiotics with friends and family if needed	30 (25.0)	87 (72.5)	3 (2.5)
9. I keep antibiotics in my cupboard for future use if needed	63 (52.5)	56 (46.7)	1 (0.8)
10. I prefer to take antibiotics when I have a cough and a cold to prevent complications	29 (24.2)	91 (75.8)	0 (0.0)
11. It is good to take antibiotics on our own without having to be examined by a doctor	13 (10.8)	105 (87.5)	2 (1.7)
12. I am worried about the impact that antibiotic resistance will have on my health and the health of my family	85 (70.8)	28 (23.3)	7 (5.8)

#### 3.1.5. Participants' scores

As shown in Table 5, participants' mean scores in all three sections of the questionnaire are above average. However, the most correct answers were given in the section concerning antibiotic consumption practices, followed by their knowledge about antibiotics and antimicrobial resistance.

**Table 5.** Questionnaire scores.

	Minimum	Maximum	Mean	Std. deviation
Knowledge about antimicrobial resistance	0	10	5.29	2.392
Knowledge about antibiotics	2	15	9.27	2.712
Practices regarding the consumption of antibiotics	1	12	8.57	2.697

Data in Table 6 shows that most of the sample had a high level of knowledge on the issues under consideration. More specifically, 63.3% of the participants had good knowledge of antimicrobial resistance and 61.7% of antibiotics, while a lower percentage was recorded on practices related to antibiotic consumption (56.7%) (diagrams 1–3). The cutoff score for categorization of participants' level of knowledge about anti-microbial resistance into high and low was 5.3, for the participants' level of knowledge about antibiotics was 9.3, and for the practices regarding the consumption of antibiotics was 8.6, according to Geta and Kibret's methodology [13].

**Table 6.** Participants' level of knowledge.

	Ν	%
Knowledge about antimicrobial resistance		
High level	76	63.3
Low level	44	36.7
Knowledge about antibiotics		
High level	74	61.7
Low level	46	38.3
Practices regarding the consumption of antibiotics		
High level	68	56.7
Low level	52	43.3

### 3.2. Inferential statistical analysis

#### 3.2.1. Comparison between groups concerning knowledge about microbial resistance, antibiotics, and practices about antibiotic consumption

According to our findings, the participants' gender has a significant impact on the formation of the score regarding their knowledge about antibiotics. Specifically, women had a statistically significantly higher score than men (9.83 vs. 8.42, p < 0.05), while no statistically significant difference was observed between the two genders regarding their knowledge about antimicrobial resistance and antibiotic consumption practices (p > 0.05). Regarding the age group to which the participants belong, there was no statistically significant difference in the scores they obtained on the questionnaires examined (p > 0.05).

The results are similar for the marital status of the participants, as the tests carried out did not reveal a statistically significant difference in the questionnaire scores between the groups examined (p > 0.05). On the contrary, the place where the participants lived appears to have an impact on the participants' knowledge about antibiotics (p < 0.05). People living in urban and semi-urban areas had higher knowledge about antibiotics compared to participants living in rural areas, as they recorded a statistically significantly higher score (10.04 and 9.40 vs. 8.40). Participants living in rural areas had the lowest mean scores on both their knowledge about antimicrobial resistance and antibiotic consumption practices.

Regarding the educational level of the participants, it was observed that individuals belonging to lower educational levels possess lower levels of knowledge regarding antimicrobial resistance, antibiotics, and their consumption practices compared to individuals belonging to higher educational levels. More specifically, in the section on participants' knowledge regarding antimicrobial resistance, it emerged that elementary/high school/lyceum graduates recorded a statistically significant difference in mean score compared to TEI/University graduates (p < 0.05). Furthermore, high school graduates had a lower level of knowledge about antibiotics compared to graduates of Higher Educational Institutions (TEI and University), while primary and high school graduates had the lowest mean score in practices related to antibiotic consumption compared to the other educational levels (p < 0.005).

Finally, the results of the tests carried out showed that the profession of the participants affected the mean score they had regarding knowledge about antibiotics. It was observed that people who declared that they are public servants had higher knowledge levels about the use of antibiotics compared to other professions (p < 0.005) (Table 7).

**Table 7.** Comparison between groups concerning knowledge about microbial resistance, antibiotics, and practices about antibiotic consumption.

	Knowledge about microbial resistance		Knowledge about antibiotics		Practices about antibiotics consumption	

	Mean ± S.D	P value (effect size)	Mean ± S.D	P value (effect size)	Mean ± S.D	P value (effect size)
Gender (N)						
Female (72)	5.54 ± 2.251	0.148* (r = −0.04)	9.83 ± 2.578	0.003* (r = −0.21)	8.88 ± 2.621	0.093* (r = −0.01)
Male (48)	4.92 ± 2.567		8.42 ± 2.712		8.10 ± 2.769	
Age group (Ν)						
19–39 (38)	5.58 ± 2.511	0.506** (r = −0.02)	9.58 ± 2.332	0.456** (r = −0.02)	8.71 ± 2.525	0.334** (r = −0.02)
40–60 (64)	5.27 ± 2.283		9.28 ± 2.908		8.77 ± 2.617	
>60 (18)	4.78 ± 2.557		8.56 ± 2.749		7.56 ± 3.222	
Marital status (Ν)						
Married (77)	5.45 ± 2.314	0.699** (r = −0,02)	9.56 ± 2.552	0.386** (r = −0.04)	8.75 ± 2.787	0.370** (r = −0.03)
Unmarried (29)	4.90 ± 2.782		8.86 ± 2.985		8.52 ± 2.262	
Cohabitating (5)	5.40 ± 2.408		9.00 ± 3.606		6.80 ± 3.271	
Divorced (9)	5.11 ± 1.833		8.22 ± 2.682		8.11 ± 2.934	
Area of Residence (Ν)						
Urban area (45)	5.87 ± 2.555	0.064** (r = 0.12)	10.04 ± 2.738	0.015** (r = −0.16)	8.60 ± 2.911	0.724** (r = 0.01)
Rural area (45)	4.76 ± 2.423		8.40 ± 2.791		8.38 ± 2.674	
Semi-urban area (30)	5.23 ± 1.924		9.40 ± 2.207		8.80 ± 2.455	
Level of Education (N)						
Primary school (9)	4.44 ± 2.068	0.001** (r = 0.29)	8.11 ± 2.934	0.001** (r = 0.28)	6.89 ± 3.723	0.020** (r = 0.20)
High school (11)	4.00 ± 2.236		7.18 ± 2.562		7.36 ± 2.873	
Lyceum (37)	4.35 ± 2.741		8.76 ± 2.302		8.16 ± 2.774	
Vocational Training Institute (14)	5.29 ± 1.729		8.57 ± 2.875		8.14 ± 2.476	
Τechnological Εducational Ιnstitute (24)	6.38 ± 1.740		10.50 ± 2.449		9.75 ± 1.962	
University (25)	6.52 ± 1.982		10.56 ± 2.501		9.40 ± 2.291	
Occupation (Ν)						
Public servant (55)	5.87 ± 2.117	0.175** (r = −0.08)	10.64 ± 2.304	0.004** (r = −0.23)	9.73 ± 1.957	0.094** (r = −0.13)
Private sector employee (19)	5.16 ± 2.566		8.68 ± 2.540		9.00 ± 1.915	
Freelancer (10)	3.80 ± 3.259		7.80 ± 2.781		7.20 ± 2.530	
Farmer (22)	4.77 ± 2.429		7.50 ± 2.304		7.05 ± 3.484	
Other (14)	5.07 ± 2.018		8.50 ± 2.534		6.79 ± 2.607	

Note: *Mann–Whitney test. **Kruskal–Wallis test.

#### 3.2.2. Correlations between knowledge about microbial resistance, antibiotics, and practices about antibiotic consumption

For the correlation test, the nonparametric Spearman's rank correlation coefficient (rs) was used, since the variables under examination did not follow a normal distribution.

Results are presented in Table 8. It was found that there is a positive correlation between the participants' knowledge about antimicrobial resistance and antibiotics and their consumption practices (p < 0.05). Specifically, the more participants' scores on their knowledge of antimicrobial resistance and antibiotic use increased, the more their scores on consumption practices increased.

**Table 8.** Correlations (r_s_) between knowledge about microbial resistance, antibiotics, and practices about antibiotic consumption.

	Knowledge about microbial resistance	Knowledge about antibiotics	Practices about antibiotics consumption
Knowledge about microbial resistance	1.000	0.449* 0.000	0.300* 0.001
Knowledge about antibiotics	0.449* 0.000	1.000	0.482* 0.000

Note: *Correlation is significant at the 0.01 level (2-tailed).

## 4. Discussion

The present study aimed to investigate the level of knowledge and attitudes of primary health care recipients regarding antimicrobial resistance and antibiotic consumption, as well as to assess misconceptions and lack of knowledge, which may constitute an obstacle to the prudent use of antibiotics and a meaningful understanding of the problem of microbial resistance.

The results of the study revealed that the participants had a relatively satisfactory level of knowledge regarding antimicrobial resistance, recognizing the impact it has on public health, and its connection with the inappropriate use of antibiotics; however, several misconceptions and gaps in knowledge about antibiotics led to the adoption of incorrect practices of antibiotic use. Specifically, more than 8 out of 10 participants said they had heard of antimicrobial resistance, while a slightly lower percentage knew that this term refers to the ability of microbes to become resistant to the action of antibiotics. A study conducted in Cyprus showed that, although a large percentage of the population had heard the term “antimicrobial resistance”, they did not have an adequate understanding of its meaning [15]. The incomplete clarification of the term may lead to an incomplete understanding of the causal relationship between the indiscriminate use of antibiotics and antimicrobial resistance [16].

The level of knowledge about antimicrobial resistance appears to have increased, leading to greater public awareness. Almost 7 out of 10 respondents recognized antimicrobial resistance as a major public health problem, both nationally and internationally, and aptly linked it to the inappropriate use of antibiotics in humans, mainly through taking antibiotics without medical advice. At the same time, they expressed concerns about the potential impacts of antibiotic resistance on their health and the health of their family members. Similar findings were recorded in a multinational study [17], where two-thirds of participants perceived antibiotic resistance as an issue that may affect them personally. In our study there was also a lack of sufficient understanding regarding the transmission of antimicrobial resistance, since half of the respondents answered that it cannot be transmitted from person to person, a finding that agrees with that of another survey [18], while the research by Michaelidou et al. [15] showed that participants incorrectly believe that only people who use antibiotics regularly are at risk of resistance.

Regarding sources of information about antimicrobial resistance, our findings demonstrate that participants mainly obtained information from television and the internet. This is a concern, as these sources may not always be reliable or scientifically substantiated, which can lead to the reproduction of misconceptions about the use of antibiotics and the development of antimicrobial resistance. In contrast, in a similar study [15], health professionals emerged as the main source of information. This differentiation between the two studies may reflect the variation in the utilization of health professionals and the reinforcement of their role as important sources of information.

In addition, most participants seemed to know that antibiotics may cause adverse effects in the human body, such as diarrhea, and fewer knew that they can affect body flora. This finding is in line with the results of a relevant survey conducted in European Union member states, according to which most participants correctly recognized that the use of antibiotics is often associated with side effects, such as diarrhea [19].

Particularly encouraging is the finding that the vast majority disagreed with stopping antibiotic treatment before its completion, even in case of improvement of symptoms, and seemed to avoid this practice, choosing to comply with medical recommendations and instructions. This finding is consistent with previous studies, which reported comparatively lower rates of individuals discontinuing antibiotic treatment based on symptom resolution [20],[21]. In contrast, in the study by Karuniawati et al. [22], approximately half of the respondents reported that they had considered discontinuing the antibiotic course once symptoms were resolved.

Regarding knowledge on the indications for administering antibiotics, approximately one out of two participants believed that any viral infection with fever should be treated with antibiotics, even though almost 7 out of 10 knew that mild infections can be resolved without taking antibiotics. The findings align with a previous study documenting similar misinformation, highlighting the widespread and erroneous belief that antibiotics can be used to treat infections and relieve fever [22]. According to surveys, the lack of sufficient knowledge seems to reinforce the tendency of patients to request antibiotics from doctors, even in cases such as the common cold, to accelerate recovery [14],[23]. In contrast, a higher level of knowledge was recorded in a relevant study conducted in Cyprus, where about one-third of participants believed that viral infections respond to antibiotic treatment [15]. This finding suggests that public awareness may vary significantly depending on the region and/or local awareness campaigns on the proper use of antibiotics. Furthermore, it is crucial to acknowledge that the ambiguity of technical terminology may have an impact on some of the misconceptions found in our results, especially in relation to the spread of AMR. Terms like “transmission” and “resistance” can be difficult for the general public to understand in many public health surveys; for instance, people frequently mistakenly think that the drug causes resistance in the human body rather than the bacteria. This implies that rather than a genuine lack of awareness, some “incorrect” answers can be the result of a misinterpretation of clinical jargon. This finding emphasizes the need for greater language and culturally appropriate health communication that breaks down difficult medical ideas in order to increase Greek people's health literacy.

Regarding antibiotic consumption practices, the findings of this study show that, while most participants stated that they did not pressure doctors to prescribe antibiotics, the overwhelming majority nevertheless believed that doctors should take the time to explain to their patients why they do or do not prescribe antibiotics. However, half of the participants reported that they had not followed medical advice and taken antibiotics without a prescription in the past, ignoring the consequences of this behavior. A previous study in Greece showed that 52.6% of participants stated that access to antibiotics without a prescription was easy, despite the legislation in force since 2020 that prohibits their sale without a prescription [14]. In contrast, a study conducted in 2021 found a significant decrease in the availability of antibiotics without a prescription, indicating that the implementation of new legislation on electronic prescribing had a positive impact on reducing these practices [24], making it more challenging for patients to get these drugs without a digital prescription. Our research indicates that the use of leftover drugs from earlier treatments is now the main cause of “self-medication”. This suggests a “normalization” of antibiotic storage, where patients see these medications not as carefully regulated, one-time clinical tools but rather as household mainstays for anticipated emergencies in the future. As a result, the challenge in the Greek primary care context has changed: it is now necessary to confront the deeply ingrained cultural practice of storing and sharing antibiotics at home rather than merely focusing on pharmacy “gatekeeping”.

Internationally, antibiotic self-administration shows significant variation. Countries such as Sweden and Norway record very low rates of over-the-counter antibiotic use [25],[26], in contrast to other studies where self-treatment rates range from 1% to 66%, depending on the methodology and demographic characteristics of the sample, as well as the national legislation about antibiotic use [24],[27]–[29]. Furthermore, a significant percentage of participants reported keeping antibiotics at home for future use, indicating established self-medication practices in the population. Similarly, storing antibiotics for future use appears to be a common practice internationally. Although some studies report low rates of this practice [23], others report rates ranging from 40% to 69% [27]–[29]. This behavior may stem from a false sense of security, in which keeping antibiotics at home ensures immediate availability in case of need, potentially bypassing the need for timely medical evaluation and guidance. The apparent discrepancy between participants' reported activities and their judged “good practices” is a noteworthy finding of this study. The high reported rates of non-prescription purchasing (56.7%) and stockpiling (52.5%) contrasted with the large percentage of respondents who indicated sentiments in favor of judicious antibiotic use. This disparity raises the possibility of a “knowledge–practice gap” or a social desirability bias, in which people give the “correct” response in principle but act hazardously in practice because of convenience, perceived urgency, or financial considerations. This phenomenon is consistent with the findings of Abuhammad et al. (2025), who pointed out that without focused, continuous intervention, high awareness does not necessarily transfer into good health habits [30].

Significant correlations emerged between knowledge about antibiotics, the gender of the participants, and the place of residence, with women having a better level of knowledge about antibiotics, while those with a higher educational level and those living in urban and semi-urban areas had higher knowledge not only about antibiotics but also about antimicrobial resistance and the correct practices of consuming antibiotics. These findings are consistent with previous research demonstrating that high educational attainment is directly associated with increased levels of knowledge about antimicrobial resistance and antibiotics and prudent antibiotic use from the public [31],[32]. Recent studies demonstrated the effectiveness of focused educational and health training programs in bridging these deficits among healthcare professionals and caregivers, despite the fact that our study found significant knowledge gaps among outpatients [30],[33]. For example, it has been shown that structured training greatly enhances mothers' awareness and perceptions in pediatric care, while our participants depended mainly on unreliable sources like television (36.7%) [30]. Our conclusion that focused public health campaigns are crucial for the Greek primary care context is supported by the impact of educational programs in specialized settings, like NICUs, which highlights the importance of ongoing education in improving practices regarding multidrug-resistant organisms [34]. Studies confirm the positive correlation between educational level and understanding of the risks associated with excessive or inappropriate use of antibiotics [15],[35],[36], whereas another research points out that besides education, the female gender and residence in urban areas are more likely to positively influence attitudes and practices regarding antibiotic use [37].

Finally, we found a positive correlation between participants' knowledge about antimicrobial resistance and antibiotics and their consumption practices, highlighting that knowledge leads to increased awareness and a sense of individual responsibility through the adoption of good practices to address the problem of antimicrobial resistance. This finding is congruent with another study's results conducted in Asir, Saudi Arabia, which showed that knowledge levels were positively associated with better practices (r = 0.141, p = 0.001) [38]. Knowledge acts as a reinforcing factor in raising awareness and a sense of responsibility toward the problem of antimicrobial resistance, strengthening the internal motivation to adopt behaviors of rational antimicrobial use. Our findings also point to the community pharmacist's position as a major lost opportunity in public health outreach. Despite being frequently the initial point of contact for patients in the Greek healthcare system, pharmacists are still underutilized as frontline educators in antimicrobial stewardship. Pharmacists should function as “gatekeepers” who offer evidence-based guidance regarding the viral basis of the majority of common colds, rather than just dispensing medication. The found knowledge gaps might be filled by strengthening the pharmacist–patient relationship through required educational modules, turning the pharmacy from a location for non-prescription acquisition into a key hub for antibiotic stewardship.

## 5. Limitations

This study has several limitations that should be mentioned. First, the study relies on self-reported data, which may introduce social desirability bias. Participants might underreport inappropriate behavior or overreport good practices toward more favorable responses. Second, the study was conducted in a specific region and at one health setting; therefore, the findings cannot be representative and may not be generalized to the public of the country. Finally, the study focuses mainly on quantitative measures of knowledge and attitudes. It may lack qualitative insights into the deeper reasons behind misconceptions or non-compliance, such as cultural beliefs, mistrust in healthcare, or economic pressures. Additionally, it is acknowledged that certain items' binary classification of “correct” versus “incorrect” answers may contain dubious presumptions. A simplistic poll might not adequately capture clinical details such as the superiority of brand-name versus generic antibiotics or the need for antibiotics for particular illnesses like ear infections. Therefore, rather than being an absolute indicator of scientific accuracy, knowledge scores should be regarded as congruent with broad public health norms.

Furthermore, this study's analysis was restricted to bivariate comparisons (Mann–Whitney and Kruskal–Wallis tests). The study was unable to adequately account for potential confounding factors across demographics and other variables due to the lack of multivariable modeling. To more clearly identify the independent variables of knowledge and practices, future studies should use logistic or linear regression models.

## 6. Conclusions

The study highlights that while primary health care recipients demonstrate a generally satisfactory awareness of antimicrobial resistance, significant knowledge gaps and misconceptions persist, particularly regarding the causes and transmission of resistance and the appropriate use of antibiotics. Although the majority recognize antimicrobial resistance as a serious public health issue, linked to the inappropriate use of antibiotics, they continue to engage in inappropriate practices such as self-medication, storing antibiotics for future use, and taking antibiotics without prescriptions. These behaviors are often influenced by unreliable information sources and sociocultural factors. Educational level, gender, and urban residency emerged as key determinants of knowledge and responsible antibiotic use. The findings emphasize the need for targeted, evidence-based public health campaigns and stronger enforcement of prescription-only regulations. Strengthening the role of healthcare professionals as primary sources of information can further promote responsible antibiotic consumption and enhance public understanding of antimicrobial resistance. Addressing these knowledge gaps is essential for fostering behavior change and mitigating the spread of resistance on both individual and societal levels.

## Use of AI tools declaration

The authors declare they have not used Artificial Intelligence (AI) tools in the creation of this article.

## References

[1] Mylona E, Kostourou S, Giankoula D (2025). Trends in antimicrobial resistance at a greek tertiary hospital over a 7-year period, including the COVID-19 pandemic. Antibiotics.

[2] Thomas F, Depledge M (2015). Medicine ‘misuse’: implications for health and environmental sustainability. Soc Sci Med.

[3] Viens AM, Littmann J (2015). Is antimicrobial resistance a slowly emerging disaster?. Public Health Ethics.

[4] Raupach-Rosin H, Rübsamen N, Schütte G (2019). Knowledge on antibiotic use, self-reported adherence to antibiotic intake, and knowledge on multi-drug resistant pathogens - results of a population-based survey in Lower Saxony, Germany. Front Microbiol.

[5] European Centre for Disease Prevention and Control, World Health Organization (2022). Antimicrobial resistance surveillance in Europe: 2020 data.

[6] Hou J, Long X, Wang X (2023). Global trend of antimicrobial resistance in common bacterial pathogens in response to antibiotic consumption. J Hazard Mater.

[7] Uddin TM, Chakraborty AJ, Khusro A (2021). Antibiotic resistance in microbes: History, mechanisms, therapeutic strategies and future prospects. J Infect Public Health.

[8] McCullough AR, Parekh S, Rathbone J (2016). A systematic review of the public's knowledge and beliefs about antibiotic resistance. J Antimicrob Chemother.

[9] European Centre for Disease Prevention and Control (2025). Antimicrobial consumption in the EU/EEA (ESAC-Net) - Annual Epidemiological Report for 2024.

[10] Frydas IS, Kouklakis E, Meletis G (2025). Genomic and phenotypic characterization of two high-risk klebsiella pneumoniae clones (ST258-blaKPC-2 and ST11-blaNDM-1) from a Greek Tertiary Hospital. Antibiotics.

[11] European Commission (2017). A European One Health Action Plan against Antimicrobial Resistance (AMR).

[12] Geitona M, Toska A, Souliotis K (2015). Antibiotic prescription practices of pediatricians and pediatric residents in hospital care in Greece. Curr Drug Saf.

[13] Geta K, Kibret AM (2022). Knowledge, attitudes and practices of patients on antibiotic resistance and use in public hospitals of Amhara Regional State, Northwestern Ethiopia: A cross-sectional study. Infect Drug Resist.

[14] Effah CY, Amoah AN, Liu H (2020). A population-based survey on knowledge, attitude and awareness of the general public on antibiotic use and resistance. Antimicrob Resist Infect Control.

[15] Michaelidou M, Karageorgos SA, Tsioutis C (2020). Antibiotic use and antibiotic resistance: Public awareness survey in the Republic of Cyprus. Antibiotics.

[16] Lajunen TJ, Sullman MJM, Baddal B (2023). Antibiotics knowledge, attitudes and behaviours among the population living in Greece and Turkey. Antibiotics.

[17] World Health Organization (2015). WHO multi-country survey reveals widespread public misunderstanding about antibiotic resistance.

[18] Drakul D, Joksimović B, Milić M (2023). Public knowledge, attitudes, and practices towards antibiotic use and antimicrobial resistance in Eastern Region of Bosnia and Herzegovina in the COVID-19 pandemic. Antibiotics.

[19] Statista (2022). Percentage of people that answered correctly when asked if the use of antibiotics often causes side-effects such as diarrhea in EU27, as of March 2022.

[20] Statista (2022). Percentage of people who got information about unnecessary consumption of antibiotics in the last 12 months in EU27, as of March 2022.

[21] Muflih SM, Al-Azzam S, Karasneh RA (2023). Public knowledge of antibiotics, self-medication, and household disposal practices in Jordan. Expert Rev Anti Infect Ther.

[22] Karuniawati H, Hassali MAA, Suryawati S (2021). Assessment of knowledge, attitude, and practice of antibiotic use among the population of Boyolali, Indonesia: A cross-sectional study. Int J Environ Res Public Health.

[23] Kamata K, Tokuda Y, Gu Y (2018). Public knowledge and perception about antimicrobials and antimicrobial resistance in Japan: A national questionnaire survey in 2017. PLoS One.

[24] Kopsidas I, Kokkinidou L, Petsiou DP (2023). Dispensing of antibiotics without prescription in the metropolitan area of Athens, Greece, in 2021—Can new legislation change old habits?. Antimicrob Steward Healthc Epidemiol.

[25] Munthe C, Malmqvist E, Rönnerstrand B (2022). Non-prescription acquisition of antibiotics: Prevalence, motives, pathways and explanatory factors in the Swedish population. PLoS One.

[26] Gravningen K, Field N, Salvesen Blix H (2020). Non-prescription purchase of antibiotics during travel abroad among a general adult population in Norway: Findings from the seventh Tromsø Study. PLoS One.

[27] Grigoryan L, Germanos G, Zoorob R (2019). Use of antibiotics without a prescription in the U.S. population: A scoping review. Ann Intern Med.

[28] Khan FU, Mallhi TH, Khan Q (2022). Assessment of antibiotic storage practices, knowledge, and awareness related to antibiotic uses and antibiotic resistance among household members in post-conflict areas of Pakistan: Bi-central study. Front Med.

[29] Wang X, Lin L, Xuan Z (2018). Keeping antibiotics at home promotes self-medication with antibiotics among Chinese university students. Int J Environ Res Public Health.

[30] Abuhammad S, Daood T, Hijazi H (2025). Evaluating the impact of a training program on mothers' awareness and perceptions of antibiotic use and antimicrobial resistance in pediatric care. BMC Public Health.

[31] Vallin M, Polyzoi M, Marrone G (2016). Knowledge and attitudes towards antibiotic use and resistance: Latent class analysis of a Swedish population-based sample. PLoS One.

[32] Pavydė E, Veikutis V, Mačiulienė A (2015). Public knowledge, beliefs and behavior on antibiotic use and self-medication in Lithuania. Int J Environ Res Public Health.

[33] Alhawatmeh H, Aljarrah M, Hweidi IM (2025). Boosting knowledge, attitudes, and practices: An experimental controlled study evaluating the effectiveness of m-health training on antimicrobial resistance for hemodialysis nurses. SAGE Open Med.

[34] Abuhammad S, Alwedyan D, Hamaideh S (2024). Knowledge, attitude, and practices of mothers working as nurses toward multidrug-resistant: impact of an educational program in neonatal intensive care unit. Infect Drug Resist.

[35] Shahpawee NS, Chaw LL, Muharram SH (2020). University students' antibiotic use and knowledge of antimicrobial resistance: What are the common myths?. Antibiotics.

[36] Sakeena MHF, Bennett AA, Carter SJ (2019). A comparative study regarding antibiotic consumption and knowledge of antimicrobial resistance among pharmacy students in Australia and Sri Lanka. PLoS One.

[37] Nepal A, Hendrie D, Robinson S (2019). Knowledge, attitudes and practices relating to antibiotic use among community members of the Rupandehi District in Nepal. BMC Public Health.

[38] Paulsamy P, Venkatesan K, Hamoud Alshahrani S (2023). Parental health-seeking behavior on self-medication, antibiotic use, and antimicrobial resistance in children. Saudi Pharm J.

